# Editorial: Etiology, Pathogenesis, and Consequences of Maladaptive Habits

**DOI:** 10.3389/fpsyg.2019.02613

**Published:** 2019-12-12

**Authors:** Leandro Fernandes Malloy-Diniz, Damien Brevers, Ofir Turel

**Affiliations:** ^1^Mental Health, Federal University of Minas Gerais, Belo Horizonte, Brazil; ^2^Addictive and Compulsive Behaviour Lab, Institute for Health and Behaviour, University of Luxembourg, Esch-sur-Alzette, Luxembourg; ^3^Department of Information Systems and Decision Sciences, California State University, Fullerton, CA, United States

**Keywords:** habits, impulsivity, addiction, inhibitory control abilities, impulse control (pathology) disorders

Maladaptive habits are behavioral patterns of inflexible behavior that reflect poor self-control and occur despite their harmful consequences. Those behaviors lead to undesirable outcomes in a broad range of a person's life domains. It is important to note that maladaptive habits, while prevalent, are not always a symptom of a disorder. Maladaptive habits develop in both psychiatric/neurological disorders and non-clinical samples; and reflect context-specific failure of self-control. There are many explanations concerning the etiology, pathogenesis, and consequences of maladaptive habits. Yet, more research is needed for understanding neurobiological and psychological mechanisms that support maladaptive habit formation and maintenance.

The study of maladaptive habits is particularly relevant since online tempting behaviors have never been so readily available and easy to engage in. Specifically, the current easy access to computers, tablets, and smartphones allows people to get repeated and continuous rewards by just pressing a button (e.g., “like,” “buy,” “bet,” “match,” “watch,” “going,” “match,” etc.). Moreover, the high volume of cues (e.g., new message notifications, and thoughts about others' thrilling experiences) makes it increasingly hard to resist the temptation of engaging in such activities.

In the light of today's high-availability of ready-to-consume rewards, the aim of this Research Topic was to integrate contributions concerning the understanding, assessment, etiology, and consequences of different types of maladaptive habits. The collection includes ten articles addressing such topics.

Considering the assessment of impulsive behavior, this Research Topic includes a study presenting the adaptation of assessment tools such as the short version of UPPS-P to a Brazilian population (Pompeia et al.). In this paper, the authors found that the psychometric properties of the Brazilian Short Version of UPPS-P support the use of a single and general impulsivity score for this scale.

Two articles addressed the issues of impulsivity and maladaptive behaviors in non-clinical samples. Gicquel et al. discussed the role of environmental and human factors (some of them related to habits) pertaining to traffic accidents in youth, and proposed strategies for preventing them. Jelihovschi et al. examined the relationship between cognitive impulsivity and decision making in professionals and students from the management field. They found that cognitive impulsiveness, related to immediatism impair complex decision-making assessed by the Cognitive Reflection Test. Together, these two studies stress the relevance of considering tempting and impulsive behavior within non-clinical samples.

Within the frame of mental disorders, the consequence of impulsivity and maladaptive habits were presented and discussed in relation to suicidal behavior (Lima et al.), attention deficits and hyperactivity disorder (Coutinho et al.), hoarding behavior (Novara et al.; Vilaverde et al.), excessive smartphone use (Khoury et al.), and feeding and eating disorders (Strangio et al.). In these articles, the authors present several patterns of hampered impulse control in individuals presenting mental health traits, suffering from mental disorders, including among their relatives. Taken together, these studies support the relationship between habits, impulsiveness, and inhibitory control deficits within a broad range of maladaptive outcomes.

Finally, the neurobiological perspective of understanding the etiology and maintenance of maladaptive habits has been adopted by a study examining the relationship between dopaminergic system and reward memory in addiction (Richter et al.). In this paper, the authors address the association between a genetic polymorphism related to the DRD2 expression (polymorphism C957T) and the memory for the reward stimulus related to addictive behavior.

Overall, the diversity of issues and knowledge fields that contributed to this topic provides unequivocal evidences that maladaptive habits are a pervasive pattern of behavior with undesirable outcomes in a large variety of settings. To a broader extent, this Research Topic offers a relevant framework for further understanding the multifactorial basis of maladaptive habits and its relationship with adverse outcomes across the lifespan and life domains, in both clinical and non-clinical samples. We hence call for more research on this topic.

## Author Contributions

All authors listed have made a substantial, direct and intellectual contribution to the work, and approved it for publication.

### Conflict of Interest

The authors declare that the research was conducted in the absence of any commercial or financial relationships that could be construed as a potential conflict of interest.

